# Editorial: Advancing plant defense: genome editing, RNAi, and synthetic biology for sustainable pest control

**DOI:** 10.3389/fpls.2026.1785705

**Published:** 2026-02-04

**Authors:** Sundararajan Balasubramani, Zhiqian Li, Ekambaram Gayathri, Dhandapani Gurusamy, Archana Singh

**Affiliations:** 1 Department of Entomology, Martin-Gatton College of Agriculture, Food and Environment, University of Kentucky, Lexington, KY, United States; 2 College of Horticulture, Northwest A & F University, Yangling, Shaanxi, China; 3 Department of Plant Biology and Plant Biotechnology, Guru Nanak College (Autonomous), Chennai, Tamil Nadu, India; 4 Department of Botany, Kongunadu Arts and Science College (Autonomous), Bharathiar University, Coimbatore, Tamil Nadu, India; 5 Department of Plant Molecular Biology, University of Delhi (UDSC), New Delhi, India

**Keywords:** artificial intelligence, CRISPR/Cas-9, nanoparticle, pest control, RNAi, synthetic biology, pesticide

The rising threat of diseases spreading through bacteria, viruses, or insects, along with abiotic stresses like drought, salinity, heat, and nutrient imbalance, is still a major limiting factor for global crop production. The major causes of crop losses due to these stresses continue to be high in the main agricultural systems, thereby putting at risk global food security in the face of climate change (Oerke, 2006; Savary et al., 2019). This situation tends to rapid shift in chemically-free, and environmentally sustainable methods of crop protection (Li et al., 2025). Synthetic pesticides have pivotal to fight against various pests and diseases, but their long, term effectiveness is now being challenged by the development of resistance, non-target toxicity, and tighter regulatory controls, hence the need for developing new and complementary strategies is essential. Currently, pesticide-based crop protection is under considerable threat from the development of resistant pest and pathogen populations, which are mainly a result of repeated and indiscriminate chemical use (Hawkins et al., 2019).

At the same time, the pollution of the environment, loss of biodiversity, and risks to human health have led to the withdrawal or restriction in the use of several widely applied agrochemicals. To make matters worse, climate change not only influences the population dynamics of pests but also allows the geographic expansion of pathogens and insects and increases the occurrence of combinations of stresses that weaken plant defense systems (Deutsch et al., 2018). Consequently, the traditional chemical solutions are gradually becoming inadequate, thus a paradigm change to new strategies of crop protection is gaining ground. At the same time, the integration of genome editing, RNA interference (RNAi), synthetic biology, and artificial intelligence (AI) has been set a strong base for future crop protection methods. Such methods by nature are highly specific, versatile, and environmentally friendly, and they thus present potent alternatives to the old chemical broad, spectrum methods (Fletcher et al., 2020). Genome editing, especially through CRISPR-Cas9 based systems, is becoming the fascinating technology for crop resistance development to various stresses. It is recognized that making genetic alterations by these precise, targeted tools is very quick and that, for instance, eliminating the genes that make the plant susceptible, improving the natural resistance, and changing the functions of genes that deal with stress can be done without the lengthy process of breeding (Zhang and Zhu, 2025).

CRISPR/Cas 9 mechanisms were used to develop crops resistant to bacterial, fungal, and viral infections in different plant species; besides that, plants were made to tolerate drought, salinity, and heat stress better. Genome edited plants can be produced without the stable incorporation of foreign DNA, which not only increases their biosafety level but also facilitates the acceptance by the public and regulatory authorities in several regions. Complementing genome editing, RNAi based technologies are also drawing a lot of attention as eco-friendly pest and disease management tool and their mechanism involves gene silencing in a sequence specific manner, thus allowing the targeted suppression of vital genes in insect pests, nematodes, and pathogens (Sheri et al., 2025).

Host induced gene silencing whereby plants are genetically modified to produce double stranded RNA (dsRNA) that targets pest genes, and spray induced gene silencing which is achieved by topical application of dsRNA, are two major RNAi delivery strategies. These methods provide the advantages of high specificity, a significant reduction in non-target effects, and a minimal disturbance of the ecosystem as compared to traditional pesticides. RNAi based crop protection technologies were initially hailed as a major breakthrough, but practical challenges have prevented their widespread adoption in open fields. The most pressing issues revolve around the degradation of dsRNA in natural environments, the limited uptake of dsRNA by target pests, and the high cost of producing dsRNA on a large scale. Researchers are continuously exploring different strategies such as nanocarrier delivery, formulations that provide protection, and choosing the best targets to maximize the effectiveness and duration of RNAi under field conditions (Koeppe et al., 2023).

Besides genome editing and RNAi, synthetic biology is rapidly gaining attraction as a versatile toolkit used for the development of next, generation crop protection strategies. It has the capability to create synthetic gene networks, reroute metabolic pathways, and tailor biological agents to manage plant stress effectively (Zhao et al., 2024). Such techniques equip plants with the ability to detect and deal with pathogen attacks or stress conditions in an innovative way instead of using constant defense mechanisms which are known to reduce growth and yield. Scientists are also engineering microbial communities and biocontrol agents that can kill pathogens, and keep plants healthy, and nutrients efficiently utilized, thus lowering the need for chemical fertilizers and pesticides.

Artificial intelligence (AI) driven systems are reshaping crop protection in various ways, such as by facilitating real, time pest and disease monitoring, forecasts with easy identification, and precision. Deep, learning models, for instance it aids farmers to make decisions on pest control measures by forecasting insect populations, determining the best time to apply treatments, and finding ways to reduce the use of chemicals through an analysis of large datasets obtained from remote sensing, sensors of fields, and historical records. Besides that, the use of AI in computational pesticide discovery is becoming mainstream, thus, the development of novel, low toxicity compounds with higher target specificity have been exponentially quickened. Phenotyping platforms with high, throughput capacity and various multi-omics technologies including genomics, transcriptomics, proteomics, and metabolomics are essential to next generation crop protection approaches (Balaska et al., 2023).

Based on profound insights of plant stress interactions, the tools could be used for the identification of the main regulatory networks and candidate targets for applications of genome editing, RNAi, and synthetic biology (Bruce, 2012). Moreover, when coupled with AI, driven data analytics, multi-omics data can support predictive, preemptive, and adaptive crop protection strategies designed for a particular agroecosystem. Although a lot has been achieved, there are still many issues that need to be solved for the effective introduction of next generation crop protection technologies at the field level. These involve the improvement of delivery systems for RNA, based products, guaranteeing the long-term stability and efficacy of engineered traits, as well as the setting up of reliable ecological risk, assessment frameworks (Bruce, 2012). The regulatory harmonization at the international level, especially for genome editing and RNAi, based products, where mismatched regulations can lead to commercialization and adoption delays. As important is the engagement of various stakeholders, including farmers, policymakers, and consumers, to build trust and to facilitate the informed acceptance of these new technologies (Kostov & Smagghe, 2025).

Six interrelated studies examining the association of molecular innovation with translational field validation and provides strong foundation for the resilience of sustainable agriculture and control insect pest. The review provides the clear insights in the manipulation of quorum sensing (bacterial communication) by bacteria integrated with the genome editing-tools for controlling the plant pathogen. The disruption of bacterial communication hinders the formation of biofilm, virulence, while enabling the CRISPR and RNAi-based technique to produce disease-resistant crops. This finding highlights the that regulatory complexity, scalability issues, and technology-transfer for large-scale field trials to achieve the lab-to-land (Anwar et al.). The contributors enlighten the implementation of foliar-applied RNAi to prevent the insect pest under field condition. Targeting the *ATP*ase genes exhibits remarkable decline in insect and pest feeding thereby optimizing the dosage and selection of target. This study integrates the laboratory outcome with real world applicability and highlights the significance of molecular diagnostics with phenotype for RNAi deployment (Govindaraju et al.).

Now a days nanotechnology is most fascinating strategy however this study focuses on formation of β-glucan nanoparticles from *Aspergillus awamori* and promotes seed germination and plant growth in tomato. These nanoparticles are a good substitute to chemicals like fungicides and reveals how the nanotechnology helps for crop improvement (Ramalingam et al.) The article provides a clear insight of crop immune responses to insect herbivores, incorporating hormonal signaling, receptor-mediated recognition, effector-triggered immunity, and metabolic reprogramming. It encompasses gene stacking and synthetic pathway engineering as pivotal strategies for attaining durable, field-stable resistance under diverse pest attack (Vasantha-Srinivasan et al.).

The review enlightens the plants exposed to unfavorable harsh conditions and pathogen attacks, have developed a remarkably complex network of RNAi pathways. They focused on explaining the connections between gene silencing and turnover, how the defective RNA degradation triggers by RNAi and importantly, it has enthralled the attention of the promising strategy for developing innovative pest control techniques (Krzyszton et al.). The study proves the universal significance of argonaute4 in viral defense mechanism underlying the CRISPR/Cas-9 mediated disruption of AGO4 in rice hoja blanca virus (RHBV). This exhibits reinforcement of conserved gene silencing mechanism in crop immunity and aid AGO4 as a target to produce antiviral crop (Ñañez et al.).

In future, moving away from reactive chemically heavy methods towards proactive, targeted, and sustainable strategies these methods can completely change farming and improve crop tolerance to biotic and abiotic stress factors. RNAi, genome editing, and synthetic biology are next generation, ecologically sound methods for plant pest control that will be combined in the future. Their integrated approaches will facilitate precision farming, environmentally friendly, effective, and pest problem, solving solutions of a long, lasting nature. These technologies will be at the core of future pest, resistant crop development, provided that suitable regulatory frameworks are set up to unlock the sustained interdisciplinary partnerships, enabling policy environments, translational research and public acceptance. Artificial Intelligence will aid to optimize RNAi, genome editing, and synthetic biology for plant pest control in the future. Together, these technologies will make pest management possible and ensure environmental safety, economic viability, and global food security.
